# Early nutrition and metabolic outcome

**DOI:** 10.1186/1687-9856-2015-S1-O14

**Published:** 2015-04-28

**Authors:** Veronica Mericq

**Affiliations:** 1 Institute of Maternal and Child Research, University of Chile, Santiago, Chile

## 

In recent years the number of extremely premature born (very low birth weight (VLBW<1500 g) children has increased as a consequence of increased survival rate. Those preterm neonates who survive have an increased risk of long-term neurological disabilities and chronic pulmonary disease. In addition, in the last two decades alterations in body composition and increased metabolic risk have been added to the list of consequences. In order to evaluate whether early patterns of infancy anthropometry and nutrition have an impact in metabolic hormonal profile and body composition we have prospectively assessed two cohorts of preterm infants and results will be presented.

